# Supplementary figures and images for: The expression of virulence genes increases membrane permeability and sensitivity to envelope stress in Salmonella Typhimurium
Source: PLoS Biol. 2022 Apr 7;20(4):e3001608. doi: 10.1371/journal.pbio.3001608 (PMC9017878; doi:10.1371/journal.pbio.3001608)

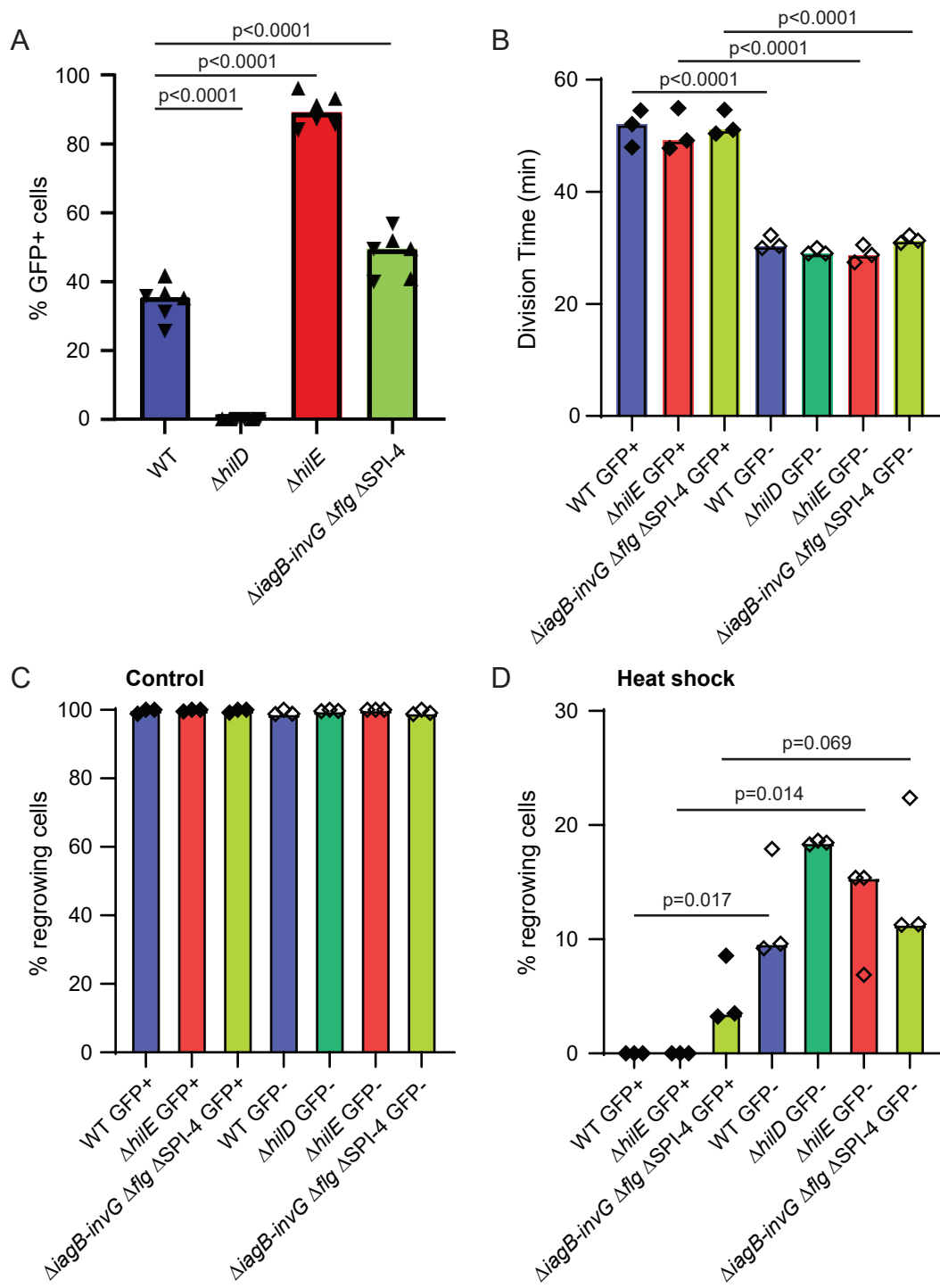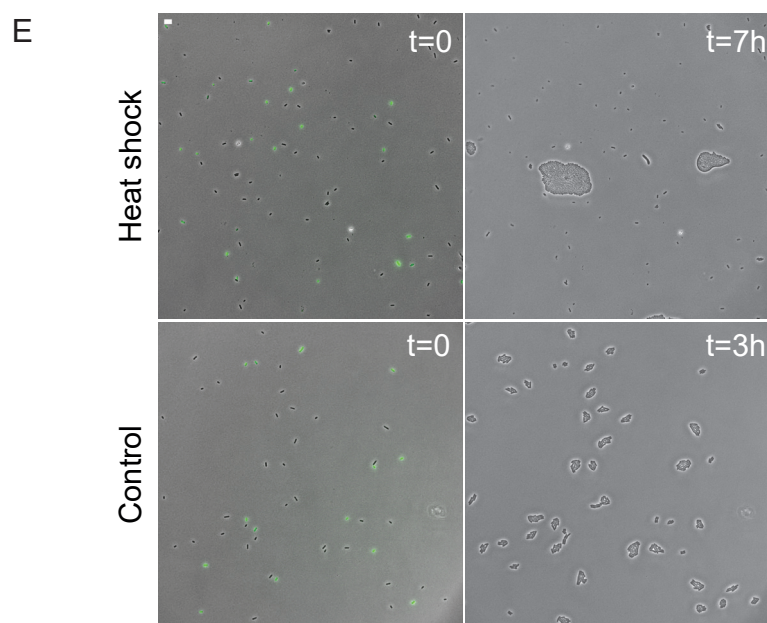

Supplement: S3 Fig — Time-lapse microscopy analysis of WT PprgH::gfp after HS (50.5°C, 15 minutes) and untreated control. The cells were observed for 12 hours posttreatment. (A) Proportion of cells expressing the HilD regulon (GFP+) from overnight cultures in LB. Triangles: cells used from control experiments, inverted triangles: cells after HS (t = 0). (B) Division time of cells expressing or not the HilD regulon from control experiments. (C, D) Proportion of cells able to grow in control conditions (C) or after HS (D). (E) Superimposed phase contrast and epifluorescence images of representative fields of view in control (upper panels) and HS (lower panels) experiments at t = 0, 3 hours or 7 hours posttreatment. Scale bar corresponds to 5 μm. p-Values were calculated using ANOVA and corrected by a Tukey HSD post hoc test. Three independent replicates. Source data are provided as a source data file (S1 Data). HS, heat shock; LB, Lysogeny broth; WT, wild-type. (PDF) [file pbio.3001608.s003.pdf]

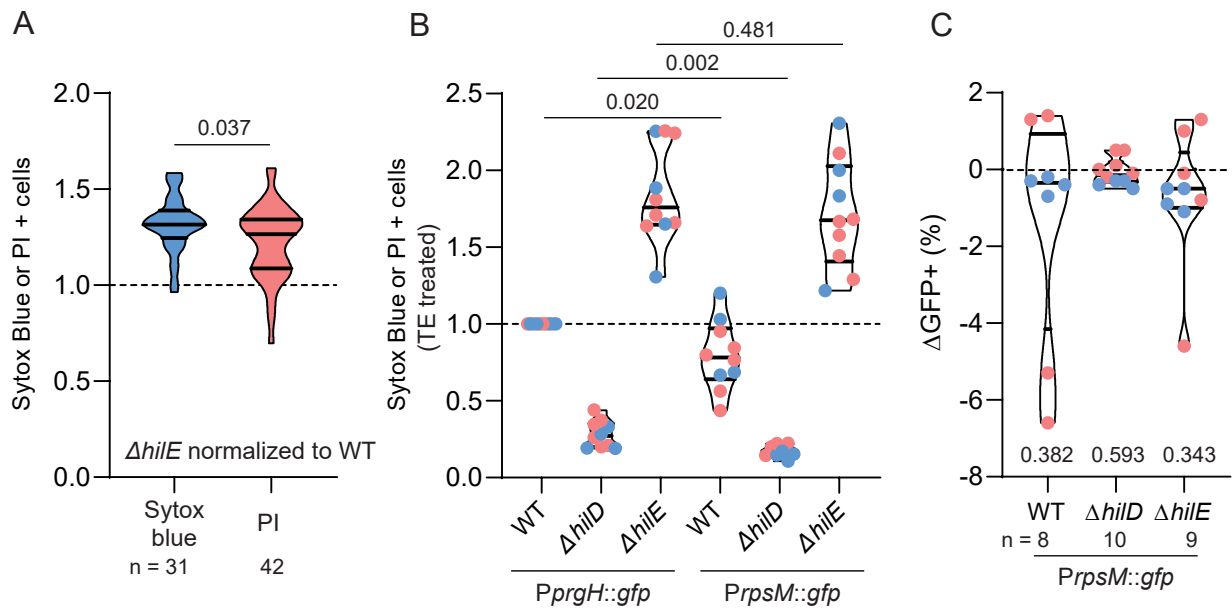

**D**

Phase contrast  
+ PI

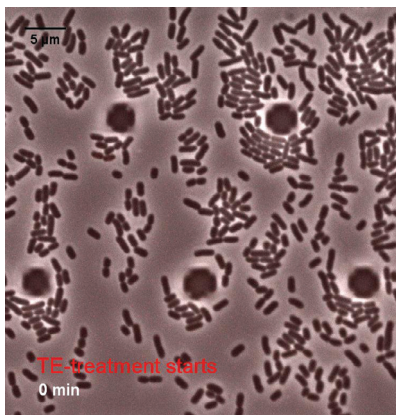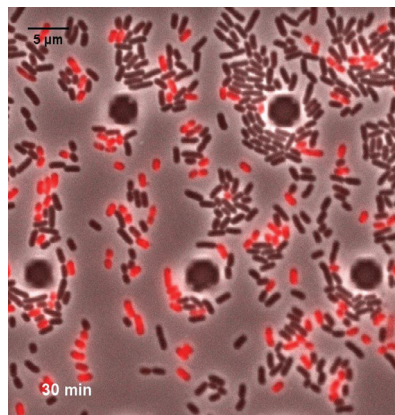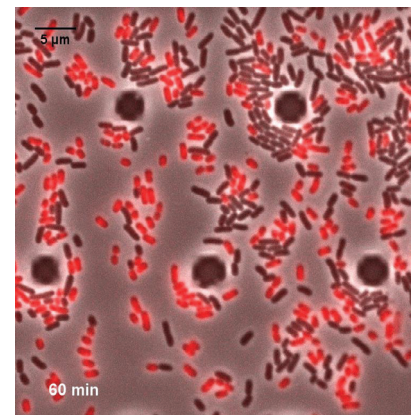

Phase contrast  
+ Sytox Blue

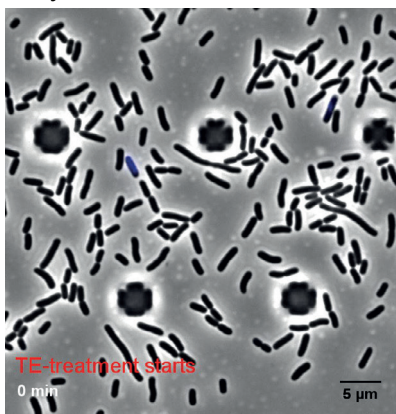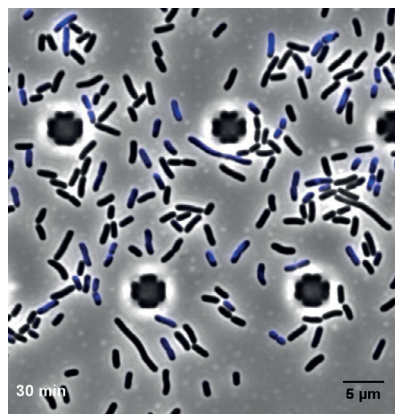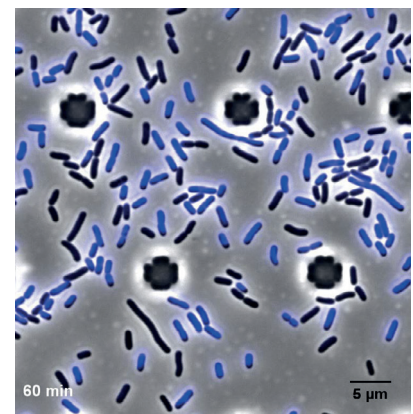

Supplement: S6 Fig — (A) Normalized frequency of dead ΔhilE reporter cells stained with either Sytox blue or PI after treatment with 100 mM Tris-10 mM EDTA. n = x indicates the number of repetitions. (B) Normalized frequency of cells stained with either Sytox blue (blue dots) or PI (red dots) after treatment with 100 mM Tris-10 mM EDTA measured by flow cytometry. The graph shows the results for WT, ΔhilD, and ΔhilE carrying the chromosomal reporter PprgH::gfp or the plasmidic PrpsM::gfp (constitutive GFP expression from pM965). The dataset is normalized by values obtained with the WT reporter strain. For comparisons against the WT, p-values were calculated using raw data in paired Wilcoxon tests. For comparisons between mutants or conditions, p-values were calculated using normalized data in unpaired Mann–Whitney tests. n = 10 repetitions. (C) Reduction of the GFP positive fraction (ΔGFP+ in percentage) among WT, ΔhilD, or ΔhilE cells negative for Sytox blue (blue dots) or PI (red dots)) treated with 100 mM Tris–10 mM EDTA compared to distilled water control. In these control experiments, the GFP was constitutively expressed (PrpsM::gfp). Significance of the deviation of the median from 0 estimated by Wilcoxon signed rank test. There was no significant loss of GFP + cells after treatment compared to control. n = x indicate the number of repetitions. In all panels, p-values are indicated within the graph below a bar marking the 2 compared conditions. (D) Imaging of WT cells by fluorescence microscopy during exposure to 100 mM Tris-10 mM EDTA (time points 0, 30, and 60 minutes) in the presence of PI or Sytox blue in a CellAsic microfluidic device. Both dyes accumulate within cells exposed to lethal stress, while almost no cells were stained at the beginning of the treatment. Source data are provided as a source data file (S1 Data). PI, propidium iodide; TE, Tris-EDTA; WT, wild-type. (PDF) [file pbio.3001608.s006.pdf]
