# Supplementary material for: The expression of virulence genes increases membrane permeability and sensitivity to envelope stress in Salmonella Typhimurium
Source: PLoS Biol. 2022 Apr 7;20(4):e3001608. doi: 10.1371/journal.pbio.3001608 (PMC9017878; doi:10.1371/journal.pbio.3001608)
Supplement: Attachment — Submitted filename: Response to reviewers.docx [file pbio.3001608.s022.docx]

**PBIOLOGY-D-21-02230R1**

**Response to reviewers**

**Editor views:**

**Reviewer #1** thinks that you don’t show evidence that the Pprg-gfp construct is directly activated by HilD, questions your presentation of the data, thinks you need to show representative images of the cell populations for all experiments, points out that over-expressing SPI-1 regulators on a plasmid is not appropriate for studying regulation in response to environmental stress, has several questions about the mouse experiment, and wants you to add more detail in the method section.

**Reviewer #2** thinks that you should add more detail in the abstract, points out several text changes for clarity, labels from figures, missing spaces, and that the strain table is lacking complete references.

**Reviewer #3** suggests another mouse experiment to test whether hilD mutants in the wild-type and triple mutant background no longer have a different phenotype in absence of intestinal inflammation, thinks that you should add additional inflammation data in the mouse experiment, and asks how important motility for intestinal colonization is.

**REVIEWS:**

Reviewer #1: Salmonella virulence, outer membrane and single cell.

Reviewer #2: Salmonella-host interactions.

Reviewer #3: Bacteria-host interactions

**Reviewer #1:** The expression of virulence increases membrane permeability and sensitivity to envelope stress in ST by Sobota et al describes experiments that address the question of fitness cost to a pathogen for expressing virulence genes. The model is Salmonella Typhimurium, and the authors explore how expression of HilD and presumably HilD-regulated genes is stressful**.** It is an interesting premise, but the manuscript is seriously flawed, not only in its writing, but in the presentation of the data.

**We would like to acknowledge reviewer 1 for this thorough evaluation of our work. Below we provide explanations, supplementary results and substantial changes in the manuscript and figures that should alleviate most criticisms and certainly strengthen this piece.**

The use of ratios and violin plots makes it very difficult to discern what is going on. Perhaps binning the results and their distribution would give a better overall picture of what is happening in the overall population. Furthermore, representative images need to be shown associated with each experiment.

**About using ratios: We correct fluorescence values (GFP, NPN) for the amount of cells in each samples as reflected by the optical density (OD). The more cells, the more signal, therefore variations in the amount of cells between samples must be normalized. This is standard procedure when measuring fluorescence in batch cultures.**

**Moreover, an important source of noise between repetitions was day to day variations in the induction level of the HilD regulon. Such variation is clearly visible in Figure 2 panel D. Small changes in media composition over the three years of work required to assemble this dataset may explain this phenomenon. This generates substantial variability in NPN uptake and stress sensitivity measurements between independent experiments. Therefore, to be able to compare mutants not always studied in parallel, we normalized the data using values from WT internal controls always performed in parallel with a given mutant or condition. A value of 1 means Fluorescence signal/OD for a given mutant equals Fluorescent signal/OD for the WT. When mutants were compared to parallel WT controls, non-normalized datasets were used to perform paired difference tests (Wilcoxon). When two mutants were compared, normalized values were used in unpaired difference tests (Mann-Whitney). This is now explained in more details in the methods section (line 634 to 650).**

**About using violin plots: Despite normalization and exclusion of outliers using the ROUT method, substantial variations remained between repetitions. Therefore, in order to be as transparent as possible about the distribution of the experimental values in each conditions, we use violin plots that provide clear information about distribution of values especially when the number of repetitions is high. However, for experiments comprising 10 or less repetitions, the points are added to the figure as per PLoS guidelines.**

**About providing representative images: Additional microscopy imaging controls are now included (Fig S4B, S5C, S6D). Note that imaging was already performed to confirm flow cytometry measurements of stress sensitivity (cf. Movie S1 and corresponding analysis in figure S7) and after heat shock treatment (figures 2 and S3).**

**Unfortunately, imaging is not an option to measure NPN uptake because NPN excitation wavelength is so low (peak between 340 and 360 nm) that microscopy setups available on the market do not allow to properly detect this dye and to perform single-cell level quantitative analysis. The problem is not only related to using non-standard filters, but to the fact that neither halogen lamps nor commercially available LED sources offer excitation below 370 nm, explaining why no NPN uptake imaging can be found in the literature.**

**This being said, while preparing this revised version, we performed experiments using the closest commonly available microscopy settings (i.e., those for DAPI staining). As expected, images could not be used to quantitatively analyze NPN uptake at the single-cell level. Nevertheless, the staining was visible in a small fraction of the cells, and this confirmed membrane localization of the dye. We provide below, for reviewers only, pictures showing few bacteria in which NPN was detected in the membrane especially after treatment with polymyxin B (the positive control used in this study).**

**NPN**

**NPN**

**Polymyxin B**

**Phase contrast**

**DAPI**

**Merge**

A

B	Deconvolved

**Figure for reviewers: NPN emits fluorescence in membranes**. **A.** Microscopy pictures of WT cells treated or not with polymyxin B and incubated 45 min in the presence of NPN. From left to right are phase contrast images (black) and NPN fluorescence detected with DAPI settings (blue), followed by merged images. **B.** Close-up of an NPN stained cell showing accumulation of NPN in the membrane (no Polymyxin treatment). The panel on the right shows the same cell after deconvolution of the image on the left.

**Method:** Microscopy pictures were taken from samples incubated 45 min with NPN. Samples were deposited onto 1% agarose pads and allowed to dry. The images were acquired using softWoRx 6.0 software with a Delta Vision Core microscope equipped with a UPlan FL N 100x objective (Olympus) and a pco.edge sCMOS camera. Due to lack of a filter set adequate for NPN detection, we used the DAPI filter set with 50% intensity and 1 sec acquisition time. Image deconvolution was performed using softWoRx 6.0. Image preparation was performed with Image J.

1. The authors employ a Pprg-gfp construct as a reporter for HilD, but they never show evidence that it is directly activated by HilD. If this has been previously shown, it should be stated explicitly and referenced. From my reading of the literature, HilD is not directly regulating prgH (ref 8), which in my view is problematic for their analysis.

**Response to Point 1: The reviewer 1 is right, previous studies have shown that HilD regulates P*prgH* indirectly via HilA (Akbar et al. Mol.Mic. 2003** [**https://doi.org/10.1046/j.1365-2958.2003.03322.x**](https://doi.org/10.1046/j.1365-2958.2003.03322.x)**). This was stated in our manuscript.**

**In our opinion there are several reasons why using P*prgH*::*gfp* as reporter for the activity of HilD is appropriate:**

**It is a standard approach to measure the activity of transcriptional regulators or sigma factors via the expression of downstream reporter genes. The reason for this is that regulators (such as HilD) are often expressed at levels that are too low for detection by fluorescence from single copy fusions inserted into the chromosome. Also, important regulators tend to be under post-transcriptional and post-translational control, so their actual regulatory activity can only be properly assessed via readout of the promoters on which they act.**

**More specifically, the regulation of HilA via HilD is tight enough to use the P*prgH*::*gfp* transcriptional fusion as a proxy for the expression of the whole HilD regulon. Two approaches presented in our manuscript confirmed this fact. First, the deletion of *hilD* abolished the expression of P*prgH*::*gfp* (Fig. S1A), showing that HilA or other regulators do not bypass HilD, as reported in Akbar et al. Mol.Mic. 2003. Second, the comparative proteome on sorted cells revealed that the entire HilD regulon was significantly more expressed in P*prgH*::*gfp* positive cells (“ON” cells) than in the OFF cells (proteomics fig. S1B and Table S1). In other words, we clearly show that the P*prgH*::*gfp* fusion is a robust reporter of the activity of HilD. This is explained in the result part lines 89 to 110.**

**Furthermore, the use of HilD mutant, the HilE mutant (HilE inhibits HilD) as well as KO’s of functions regulated by HilD (e.g. T3SS-1 and flagella) further validate the cost generated by the expression of the HilD regulon, independently from the actual P*prgH*::*gfp* readout.**

2. Why is the data always presented as a function of OD? It makes it hard to understand what is actually being plotted. If every sample is at the same OD, then why don't the authors present the data simply as relative fluorescence units? If all the samples are n=7, this does not need to be presented on the graph. It should be included in the legend. What do the numbers in the figure indicate? I can't find this in the legend. What was the level of autofluorescence and how was it corrected? P values for figures should NEVER be listed separately in SI. In Figure 1B, why is there so much more variation in the hilE strain compared to the GFP?? Using a control that expresses GFP on a plasmid does not evenly compare copy numbers. I would like to see an image of the membranes of the two populations, those expressing HilD and those that show reduced NPN partitioning. It isn't clear how reduced uptake of NPN is correlated with a reduction in membrane integrity, and the authors have not made this link clearly. For all experiments, representative images of the cell populations should be shown.

**Response to Point 2: Please see our response above explaining the necessity to normalize the fluorescence signal in batch cultures according to the optical density. We have included the number of repetition under each conditions as per PLoS guidelines. Numbers above horizontal bars in the figures are p values, this is now explained in all figure captions. For clarity, mentions of correction for the OD were removed from axis labels.**

*“What was the level of autofluorescence and how was it corrected?”*

**The level of bacterial auto-fluorescence corrected for the OD is now sustracted from NPN uptake measurements (cf. revised figures 1, S2). The fluorescence due to bacteria in the absence of NPN in the medium was low enough to not alter our conclusions.**

*“P values for figures should NEVER be listed separately in SI.”*

**P values discussed in the main text are now visible on the figures. We however chose to keep supplementary tables with additional statistical analyses in case some readers would be interested to look at different comparisons they may find relevant.**

*“In Figure 1B, why is there so much more variation in the hilE strain compared to the GFP??”*

**Some points indeed presented NPN uptake values not correlating with the proportion of cells expressing the HilD regulon in some repetitions, although not deviating enough from the other repetitions to be excluded by the outlier ROUT method. Noise in experimental measurement is sometimes difficult to explain and in these cases, we must admit we do not know. The essay was nevertheless robust enough to allow significant differences to be detected between strains and conditions.**

*“Using a control that expresses GFP on a plasmid does not evenly compare copy numbers.”*

**Plasmid copy number can indeed change from cell to cell. However, this does not affect the validity of the control in Figure 2. Indeed, the point was to demonstrate that a strong *gfp* expression did not influence NPN uptake readout. This is why we used a constitutive *gfp* expression from a multi-copy plasmid ensuring sufficiently strong and homogenous fluorescence signal. A supplementary panel has been added to figure S2 showing the GFP distribution in cells carrying pM965 (unimodal) compared to the P*prgH*::*gfp* signal (bimodal).**

*“I would like to see an image of the membranes of the two populations, those expressing HilD and those that show reduced NPN partitioning.”*

**We believe reviewer 1’s main concerns come from the fact that we do not directly link the expression of the HilD regulon with NPN uptake at the single cell level like this was done for stress resistance in this study. We have discussed the technical limitation of microscopy imaging to analyze NPN membrane staining in our response above.**

**However, we would like to insist on several points: i) NPN uptake is a well-established proxy for membrane permeability in *Salmonella* (Helander and Mattila-Sandholm J.A.M 2008,** <https://doi.org/10.1046/j.1365-2672.2000.00971.x>**), ii) We show a clear correlation between NPN uptake and the expression of *hilD* at the population level (fig. 1B), iii) Deleting functions upregulated by HilD reduce both NPN uptake and stress sensitivity (Tris EDTA and heat shock). Overall, this are converging evidence that the membrane of HilD expressing *S.*Tm cells is disturbed.**

**Looking at the membrane of cells expressing *hilD* or not in the WT context is in principle possible using correlative light-electron microscopy (CLEM) with the P*prgH*::*gfp* reporter. However this technique remains challenging and not readily available in our institute.**

**In general, providing direct single-cell level measurements of membrane integrity is not trivial. Because of this, we rather focused on single-cell level analysis of stress sensitivity and its potential consequence for virulence evolution during infections *in vivo*.**

*“It isn't clear how reduced uptake of NPN is correlated with a reduction in membrane integrity, and the authors have not made this link clearly.”*

**As discussed above, we did not make this link because our data actually suggest the contrary, i.e., increased uptake of NPN correlates with an increase in sensitivity to membrane stress (heat, bile, Tris-EDTA), so supposedly, an inherent reduction in membrane integrity in cells of *Salmonella* expressing the HilD regulon.**

**This is in line with previous observations that NPN passage through the LPS of the outer-membrane and its accumulation in the hydrophobic membrane leaflets happen for instance when aminoglycosides act as a divalent cation binding site on the *P. aeruginosa* outer membrane (Loh et al. AAC. 26 1984 546 551** [**https://doi.org/10.1128/AAC.26.4.546**](https://doi.org/10.1128/AAC.26.4.546)**) or in *E. coli* treated with the antimicrobial peptide indolicidin (Falla et al. JBC Vol. 271, No. 32, 9, pp. 19298–19303, 199** [**https://doi.org/10.1074/jbc.271.32.19298**](https://doi.org/10.1074/jbc.271.32.19298)**).**

**This is now explained in the main text line 158 to 162.**

*“For all experiments, representative images of the cell populations should be shown.”*

**Images are now provided for all staining procedures except for NPN as explained above.**

3. Figure 1D/E are described before Fig. 1C, which was never described.

**Response to point 3: Thank you for noticing this oversight from our part. This has been corrected (line 128).**

4. The results section begins with all data that is in Supplementary information. This does not make the manuscript easy to read or straight forward. The construction of the manuscript and its presentation style is difficult. The tables in Supplemental information are not well annotated. The authors need to distill the essential features of the table, put it in the regular manuscript and then have the full table in SI.

**Response to point 4: We think it is important to start with a brief description of the tools used to study the pattern and cost of HilD regulon expression (i.e., the transcriptional fusion P*prgH*::*gfp*). As discussed above, the proteomic analysis on sorted cells was important to validate that this construction was indeed a reliable proxy for the expression of the whole HilD regulon. However, the P*prgH*::*gfp* construction and the HilD regulon being already well-characterized (Hautefort et al. AEM 2003; Martinez-flores et al. Sci Rep. 2016. doi: 10.1038/srep37858), nothing unexpected emerged from these experiments, which is why we decided to present the data as supplementary material.**

**This being said, we understand that this choice makes the manuscript not straightforward. Therefore, as suggested by reviewer 1, a table was included in the main text (Table 1) that focus on the expression of multi-protein complexes controlled by HilD in sorted cells expressing the P*prgH*::*gfp* fusion.**

**We would like to apologize for the lack of information regarding the supplementary tables. This is now improved with captions for each table. Table S1 has been substantially modified to help the reader understanding which functions are controlled by HilD.**

5. The way the data are plotted seem to obscure the single cell differences. Are there two distinct populations that are responding? And if so, why not separate them and analyze them separately? The differences would be more compelling.

**Response to point 5: Regarding figures 1, 2C-E and 3, where fluorescence from GFP, NPN, PI or Sytox is measured in batch culture or by flow cytometry, each point represents either fluorescent signal or a proportion of cells in a population for a given repetition.**

**The proportion of HilD expressing cells (ON) is revealed by the GFP from P*prgH*::*gfp*. This proportion of ON cells before treatment correlates with the amount of NPN accumulating in a population of cells and with the proportion of cells stained by PI or Sytox after lethal treatment by Tris/EDTA.**

**As reviewer 1 suggests by “*separate them and analyze them separately*”, it was possible to measure the reduction in GFP positive cells (HilD regulon “ON”) in the surviving fraction after lethal treatments using either microscopy imaging or flow cytometry (figure 2A, F and G).**

**Sensitivity to stress in the ON cells was confirmed by microscopy imaging, cf. movie S1, with PI staining during Tris/EDTA treatment. The ON cells are clearly dying from stress and this was indeed compelling. However, the low throughput of imaging did not allow testing multiple conditions, sufficient repetitions and various mutants as with plate reading and flow cytometry analysis.**

**Physically sorting HilD ON from OFF cells by FACS before treatment is not an option as it takes several hours to isolates enough cells and requires to keep the cells on ice, thus making screening of mutants, conditions, and repetitions, impractical and not compatible with further stress test analysis.**

6. Over-expressing SPI-1 regulators on a plasmid is not appropriate for studying regulation in response to environmental stress, especially when expression is not homogeneous.

**Response to point 6: Depending on the origin of replication, the plasmid copy number may vary from cells to cells therefore complicating the study of response to environmental stress. However, this is not relevant with respect to the use of p*hilA* in our work.**

**Indeed, in this study, the over-expression of HilA controlled by the arabinose inducible promoter P*bad* from a multi-copy plasmid allows robust decoupling of virulence expression from environmental regulatory signals in a vast majority of cells. The revised figure S10 shows that this system yield a clearly dominant ON population that remains ON after sublethal TE treatment. In our case this was necessary and sufficient to: i) study the effect of deleting genes regulated by HilD via HilA (figures 1D,E and 2H,I), and ii) demonstrate that decoupling virulence expression from negative regulation by environmental cues was indeed detrimental to *Salmonella* when conditions became lethally stressful for the ON cells (figure 3C and D).**

7. The authors try to make the **correlation between NPN sensitivity and TE sensitivity** **with respect to ΔSPI-1 and Δflg strains**, but the results do not compare when looking at Sytox or PI staining of dead cells (Fig. 2H-I and Fig. 1E). This would seem to negate their hypothesis that pathogenesis creates membrane stress and sensitivity to environmental stress.

**Response to point 7: We do not think we have the same opinion as the reviewer about the correlation between NPN uptake and stress sensitivity with respect to ΔSPI-1 and Δ*flg* strains. Indeed, data in panels 1D and S2A (NPN uptake) show the exact same pattern than data in panels 2H and S9A (killing by Tris/EDTA) (graphs reproduced below).**

**This is quite remarkable, given the two very different assays and readouts, and certainly in line with our hypothesis that virulence expression permeabilizes the membrane and increases stress sensitivity for the same reasons.**

**However, the differences between mutants were more pronounced for stress resistance than for NPN uptake, which is due to the higher sensitivity of the stress resistance assay. Therefore, removing the *flg* operon was enough to phenocopy Δ*hilD* or ΔSPI-1 (in which *hilD* is also removed) with respect to NPN, but not to stress resistance. Removing *iagB-invG*, SPI-4 and *flg* increased stress resistance to a level clearly closer to Δ*hilD* or ΔSPI-1. This may show that other functions slightly contribute to reduce stress resistance, but does not negate our hypothesis. This is now discussed in the manuscript, lines 258 to 262.**

**Upper left: Figure 1D ; Upper right: figure 2H ; Lower left: Figure S2B ; Lower right: Figure S9A**

8. In the mouse experiment, the triple mutant does better, but by day 3, the difference is very small. One wonders what would have happened on day 4. Why were differing numbers of mice (only 2 WT and 3 hilD- ?) used? And there is very little difference in inflammation, why? The legend is poorly explained and so the figure is difficult to interpret.

**Response to point 8: First, we would like to pinpoint that the competitive index values are presented on a base-10 logarithmic scale. An unbiased statistical analysis confirmed that the observed fitness differences are substantial.**

**We were following strict ethical rules not allowing to prolong the experiments after 3 days post-infection. Nonetheless, one can speculate that at day 4 p.i. the differences should become less pronounced. Indeed, killing of *Salmonella* is mainly mediated by a limited pool of polymorphonuclear neutrophils migrating into the gut lumen. This peaks at day 2 p.i., generating a bottleneck in the S.Tm population followed by regrowth. There is less stress from day 3 onward (as described in Maier et al. PLoS Pathog 2014 10(12): e1004557. https://doi.org/10.1371/journal.ppat.1004557). Such reduced stress burden after initial full blown inflammation could also reduce the positive effect of the triple deletion (Δ*iagB-invG* Δ*flg* ΔSPI-4) increasing stress resistance but not preventing the growth defect in HilD+ cells.**

**Along those lines, we present a new dataset (figure S12) where mice are infected with avirulent strains unable to trigger inflammation. In these conditions, expressing or not T3SS-1, flagella and SPI-4 does not make any difference when competing HilD+ vs HilD- strains. Only the growth defect associated with HilD expression allows the HilD- strains to win the competition (figure S12C). This is now discussed lines 308 to 324.**

*Why were differing numbers of mice (only 2 WT and 3 hilD- ?) used? And there is very little difference in inflammation, why? The legend is poorly explained and so the figure is difficult to interpret.*

**There seems to be a misunderstanding therefore we apologize for not being clearer in our presentation of the data in figure 4.**

**We performed competitions between two strains (HilD + vs HilD -) in different genetic backgrounds (with or without *iagB-invG* *flg* SPI-4). 5 mice were infected with a mix WT (HilD +) vs *ΔhilD* (HilD -) and 6 mice were infected by a mix Triple Mutant (HilD +) vs *ΔhilD* Triple Mutant (HilD -) at a 1:1 initial ratio.**

**The black and red dots in figure 4 are data from mice infected by the same mix 1 WT (HilD +) vs *ΔhilD* (HilD -). The only difference is that “red dot” mice were more inflamed at day 1 p.i than “black dot mice”.**

**As noticed by the reviewer, all mice were indeed inflamed at day 2 and 3 because all mice received a virulent helper WT strain to trigger comparable disease.**

**We have improved the description of the competition experiments in the main text (line 289 to 324) and in figure 4 and S11 and S12 captions.**

9. The method section is very poorly written and lacking in detail.

**Response point 9: We would like to apologize for that. We have thoroughly revised the method section and hope that it now meets the expectations of reviewers.**

Minor:

1.Lines 39-41 The authors imply that the relationship between environmental stimuli and the regulation of virulence gene expression is not understood. This statement is way overblown.

Lines 51-52 the authors state: However, the fitness-cost of virulence at the single-cell level and the facultative intra-cellular lifestyle of S .Tm strongly constrain the expression of the HilD regulon. This sentence is misleading, what turns off HilD is not the fitness cost, it is the acidic environment of the vacuole, that also directly stimulates the SsrB regulon and SPI-2 gene expression (which should be associated with a different fitness cost). This is implied but not well stated in lines 56-68. References here are out of date and not comprehensive. In the subsequent paragraph, the other key two-component systems (EnvZ-OmpR and SsrA-SsrB) systems are completely ignored.

**Response to Minor point 1. Our introductory statement underscored that we do not know the evolutionary pressures selecting for well-described regulatory mechanisms shutting down the HilD regulon upon stress outside the SCV. That is why we initially considered the intra-cellular induction of SPI-2 out of the scope of our introduction. This is now corrected with more references as suggested by the reviewer (lines 56 to 62 and 69 to 73).**

2. Embedding the figure legends in the text was a super pain to read and disrupts the flow.

**Response to Minor point 2. This is now corrected.**

3. The Introduction reads more like a grant proposal than a manuscript submission and raises a number of questions, but doesn't clearly describe their hypothesis and how they will test it.

**Response to Minor point 3. We have improved the introduction to better explain how we test our hypothesis** **and how the hypothesis was derived (line 75 to 84).**

4. Line 263 is enable supposed to be unable?

**Response to Minor point 4. This is corrected.**

5. Fig. 2B is too small.

**Response to Minor point 5. We have increased the panel size.**

6. As an example of writing, for example, instead of stating "lesser resistance to stress" say increased sensitivity to stress-the direct is more clear than the indirect. Examples of this kind of writing are present throughout the ms.

**Response to Minor point 6. Changes all over the manuscript improve the writing style.**

7. What does 1ml of 4h day cultures mean (line 471)?

**Response to Minor point 7. “4h day cultures” means late exponential phase cultures. This is now corrected.**

**Reviewer #2**: This is an interesting manuscript dissecting the envelope stress induced in Salmonella by the coordinated expression of primarily the SPI1 T3SS and flagellar apparatus. The experiments are thorough and well controlled. Interestingly, they distinguish between envelope stress and a general growth defect induced by HilD expression. My comments focus on presentation and grammar.

**We would like to thank reviewer 2 for helpful suggestions to improve our manuscript.**

Line 21. "…could allow the identification and modulation of ecological factors…"

The abstract could provide more detail of the results, explicitly the points made in the summary paragraph above.

**Response: The abstract was modified accordingly.**

Line 85. Delete "ectopic". More importantly, state where this fusion is the chromosome, emphasizing that it does not disrupt SPI1 T3SS expression.

Line 114. Replace "it" with "GFP". As it is now written, the reader is not sure if "it" refers to GFP or HilD.

Line 144. In several figure legends, you refer to "day cultures". I do not know what that means.

Line 178. You use the term "polarization" to mean maintenance of the proton motive force. Be more explicit.

Line 209. You can delete the line "Thoroughly filtered media…"

Line 263. I think you mean "able" not "enable".

**Response: We agree on all suggested corrections and modified the text accordingly. “Day cultures” was replaced by “late exponential phase”, “Polarization” was replaced by “membrane potential” measured by DiOC2(3) and DiSC3(5).**

Figs 2D, 3A, and 3C. I found the labels on the y axis to be very confusing. I thought you were measuring the fraction of GFP+ cells that were alive. I finally figured out it was the fraction of GFP+ cells. Maybe just remove the word "alive".

Fig 3 C and D. It might be useful to move the "sub-lethal" pluses and minuses above the names. With the slanted names, it is a little difficult to know if the plus or minus refers to the start of the name immediately above it or the data directly above it. It's confusing to the eye.

**Response: Figures have been improved as suggested.**

You are missing spaces in many places: before parentheses and between numbers and units. 2mg versus 2 mg, for example.

Your strain table is lacking the complete references.

**Response: These mistakes are now corrected**

**Reviewer #3:** With this paper the authors extend our understanding of fitness trade-offs of pathogenic bacterial virulence expression on the example of Salmonella Typhimurium. The senior author's previous work on this topic has progressed the field considerably, and the key findings in this work in Salmonella are likely relevant also for other pathogens. In Salmonella the HilD regulon regulates many virulence factors and other genes, including the expression of the SPI1 type 3 secretion system (T3SS-1), SPI4, and the flagella system that are all key virulence factors mediating intestinal inflammatory pathology which modifies the pathogens' intestinal niche to its own relative advantage. However, virulence expression regulated by HilD is costly and drastically slowing growth of virulence expressing bacteria, threatening the stability of virulence in Salmonella by selection of avirulent mutants. As shown in the senior author's elegant previous work, this is prevented by the bimodal expression of the HilD regulon, which counters the out-competition of the pathogen by genetically avirulent "cheater" mutants.

In the present paper, inspired by the fact that several key virulence factors, T3SS-1, flagella apparatus, and SPI4 type 1 secretion system under HilD control are major membrane complexes that may impose cell envelope stress, the authors elucidate the contribution of envelope stress to the cost of virulence expression. Single cell-based analyses provide evidence that the HilD controlled expression of virulence inflicts envelope stress (manifesting in increased outer membrane permeability, decreased heat hock survival, increased membrane depolarization and reduced viability in response to EDTA treatment stress) compared to HilD-deficient mutants. Triple deficiency for SPI1 type 3 secretion apparatus, flagella and SPI4 rescued this stress phenotype, whereas overexpression of T3SS-1 and SPI4 exacerbated it.

Sublethal envelope stress also downregulated HilD regulon induction (with was known before) and consequently increases stress resistance to subsequent lethal envelope stress.

A very interesting novel finding is that by triple-deficiency for T3SS-1, flagella, and SPI-4 in a HilD proficient genetic background, the envelope stress-related cost of virulence expression can be uncoupled from the slow-growth phenotype of the HilD regulon expressing Salmonella sub-population. The mechanistic nature of the slow growth-related cost of virulence expression remains in the dark. However, the authors conclude the study with an elegant competitive colonization experiment in the streptomycin-pretreated mouse model, showing that the envelope stress related cost of virulence expression does contribute to the growth disadvantage of virulence expressing Salmonella specifically in stressful environment of the inflamed gut.

This work is of very high quality. The manuscript is very well written and referenced. The introduction provides the relevant background and leads over to the main hypothesis in an easily understandable manner. The in-vitro experiments are very well designed, very well controlled and very carefully interpreted and adequately support the authors' conclusions. In the discussion section, the findings are rigorously and critically discussed and put in context with the current literature.

**Response: We are happy that our work has been so well understood and received by reviewer 3.**

The only point I would regard worthwhile addressing with additional experimentation regards the final mouse experiment:

1. If the authors' conclusions were correct, hilD mutants in the wild-type and triple mutant background should no longer have a different phenotype in absence of intestinal inflammation. An additional animal experiment could therefore be tried to test this prediction, using an avirulent mutant as helper strain, and the use competing test strains in a SipBC translocon deficient mutant background that is intestinally avirulent but still able to express a fully assembled T3SS-1 apparatus. (I understand that the required additional strain construction may not be trivial, and the authors may be able to provide an alternative, perhaps even better approach to address this point.)

**Response to point 1:** **This is indeed an important control. In the absence of inflammation, only the growth defect should provide an advantage to Δ*hilD* mutants. Meaning that the triple deletion *iagB-invG* *flg* SPI4 should not change the competitive advantage of HilD- strains.**

**The fact that T3SS-1 plays a marginal role in increasing stress sensitivity in the wild-type context (cf. figure 2, no significant effect of the *iagB* to *invG* deletion) allows us to use well-characterized Δ*invG* Δ*ssaV* avirulent mutants (T3SS-1 and -2 ineffective) to perform competitions in the absence of inflammation.**

**Results from these additional experiments are presented in figure S12. As expected, avirulent HilD- strains outgrow HilD+ strains at the same pace with or without *iagB-invG* *flg* SPI4. Note that this pace is rather slow compared to experiments performed with virulent strains (fig. 4). This is because the inflammation kills part of the *Salmonella* population (Maier et al. PLoS Pathogen 2014 11 (7), e1005047) while accelerating outgrowth of HilD- strains during re-growth. Accordingly, one can also observe the whole population size of *Salmonella* decreasing by day 3 p.i. because of competitive exclusion by the re-growing intestinal flora in absence of inflammation. This is presented lines 308 to 324.**

2. Since it is important here that the degree of inflammation is comparable between the two animal groups, the inclusion of additional inflammation data, such as cecal histology data, would be an improvement. Lipocalin-2 quantification is a proven quantitative readout but can be variable between experiments. The histological picture on day 1 may even more convincingly support the claim that the two mice labelled in red are indeed particularly severely inflamed.

**Response to point 2: Lipocalin-2 (LCN2) quantification is the only way to monitor inflammation without terminating the mice, which was essential here as we performed 3-days infections in order to follow the kinetics of competitions. It is therefore impossible to show histological pictures on day 1 as it would require to prematurely terminate the mice.**

**New supplementary data in figure S12 show the baseline LCN2 concentration from non-inflamed mice (roughly 1 ng/g feces). This is about 10 times lower than in less-inflamed mice at day 1 post infection with virulent strains (fig. 4). Fully inflamed mice show LCN2 concentrations 10.000 higher than the baseline. This high dynamic range makes detection of intermediate levels of inflammation via fecal LCN2 dosage fairly reliable.**

**Nevertheless, we agree that correlating inflammation with competitive index at day 1 p.i. solely based on LCN2 values in 5 mice (cf. red vs black dots in figure 4) is not the strongest argument to support our hypothesis. New data in non-inflamed mice are now provided (described above) that strengthen this point. The manuscript was modified accordingly (lines 308 to 324).**

3. Minor comment: triple mutant and wild-type background differ in motility. How important is motility for intestinal colonization?

**Response to point 3: Motility plays at least two roles during intestinal colonization: i) motility helps *Salmonella* to reach the intestinal mucosa and to trigger the disease by invading the epithelial cells (Stecher et al. IAI 2004** <https://doi.org/10.1128/IAI.72.7.4138-4150.2004>**), i.e., non-motile or chemotaxis mutants are less virulent; and ii) motility allows *Salmonella* to fully benefit from the inflammation by swimming toward the intestinal mucosa were nutrients are more abundant (Stecher et al. Cell Mic 2008** https://doi.org/10.1111/j.1462-5822.2008.01118.x**). Although, this advantage was not sufficient to exclude non motile mutants by motile helper strains in the inflamed mice (cf. figure S11). We mention this point in our discussion (lines 391 to 394).**
